# Efficacy and safety of Privigen® in patients with chronic inflammatory demyelinating polyneuropathy: results of a prospective, single-arm, open-label Phase III study (the PRIMA study)

**DOI:** 10.1111/jns5.12017

**Published:** 2013-06-19

**Authors:** Jean-Marc Léger, Jan L De Bleecker, Claudia Sommer, Wim Robberecht, Mika Saarela, Jerzy Kamienowski, Zbigniew Stelmasiak, Orell Mielke, Björn Tackenberg, Amgad Shebl, Artur Bauhofer, Othmar Zenker, Ingemar S J Merkies, on behalf of the PRIMA study investigators

**Affiliations:** 1 Reference Center for Rare Neuromuscular Diseases, Hôpital Pitié-Salpêtrière and University Paris VIParis, France; 2 AZ St-LucasGent, Belgium; 3 Department of Neurology, Universitätsklinikum WürzburgWürzburg, Germany; 4 UZ LeuvenLeuven, Belgium; 5 Department of Neurology, Helsinki University Central HospitalHelsinki, Finland; 6 Dolnośląski Szpital SpecjalistycznyWrocław, Poland; 7 Samodzielny Publiczny Szpital KlinicznyLublin, Poland; 8 CSL Behring GmbHMarburg, Germany; 9 Department of Neurology, Philipps UniversityMarburg, Germany; 10 Spaarne Hospital, Hoofddorp and Maastricht University Medical CentreMaastricht, The Netherlands

**Keywords:** chronic inflammatory demyelinating polyneuropathy, INCAT, IVIG, PRIMA, Privigen®

## Abstract

This prospective, multicenter, single-arm, open-label Phase III study aimed to evaluate the efficacy and safety of Privigen® (10% liquid human intravenous immunoglobulin [IVIG], stabilized with l-proline) in patients with chronic inflammatory demyelinating polyneuropathy (CIDP). Patients received one induction dose of Privigen (2 g/kg body weight [bw]) and up to seven maintenance doses (1 g/kg bw) at 3-week intervals. The primary efficacy endpoint was the responder rate at completion, defined as improvement of ≥1 point on the adjusted Inflammatory Neuropathy Cause and Treatment (INCAT) disability scale. The preset success criterion was the responder rate being ≥35%. Of the 31 screened patients, 28 patients were enrolled including 13 (46.4%) IVIG-pretreated patients. The overall responder rate at completion was 60.7% (95% confidence interval [CI]: 42.41%–76.43%). IVIG-pretreated patients demonstrated a higher responder rate than IVIG-naïve patients (76.9% vs. 46.7%). The median (25%–75% quantile) INCAT score improved from 3.5 (3.0–4.5) points at baseline to 2.5 (1.0–3.0) points at completion, as did the mean (standard deviation [SD]) maximum grip strength (66.7 [37.24] kPa vs. 80.9 [31.06] kPa) and the median Medical Research Council sum score (67.0 [61.5–72.0] points vs. 75.5 [71.5–79.5] points). Of 108 adverse events (AEs; 0.417 AEs per infusion), 95 AEs (88.0%) were mild or moderate in intensity and resolved by the end of study. Two serious AEs of hemolysis were reported that resolved after discontinuation of treatment. Thus, Privigen provided efficacious and well-tolerated induction and maintenance treatment in patients with CIDP.

## Introduction

The PRIMA (Privigen® Impact on Mobility and Autonomy) study was designed to investigate the efficacy and safety of Privigen in the treatment of patients with chronic inflammatory demyelinating polyneuropathy (CIDP).

CIDP is an acquired presumed autoimmune disorder of the peripheral nervous system *(Koller et al*., 2005*)* with a prevalence of 3–9 individuals per 100,000 adults, according to different studies *(Laughlin et al*., 2009*)*. CIDP is characterized by predominantly symmetrical muscle weakness, often accompanied by impaired sensation and absent or diminished tendon reflexes, leading to reduction in daily activities and quality of life expectations *(Koller et al*., 2005; *Merkies et al*., 2010a*)*. The clinical course is chronic progressive or relapsing, the latter more common in young adults *(Koller et al*., 2005*)*. Electrodiagnostic tests are mandatory for diagnosis; major features suggesting a diagnosis of CIDP are listed in the European Federation of Neurological Societies/Peripheral Nerve Society (EFNS/PNS) Guideline *(Joint Task Force of the EFNS and the PNS*, 2010*)*.

CIDP is commonly treated with corticosteroids or intravenous immunoglobulin (IVIG) *(Hughes et al*., 2008; *Joint Task Force of the EFNS and the PNS*, 2010*)*. A recent study has compared the efficacy of both in the induction treatment of CIDP *(Nobile-Orazio et al*., 2012*)*. After several randomized trials showing only a short-term improvement *(Vermeulen et al*., 1993; *Hahn et al*., 1996; *Thompson et al*., 1996; *Mendell et al*., 2001*)*, a randomized, placebo-controlled study in a large cohort of patients treated with IVIG was recently conducted, referred to as the ICE study (10% caprylate-chromatography purified IVIG [IVIG-C] in CIDP efficacy). The ICE study has demonstrated both short- and long-term improvements in disability, as assessed by the adjusted Inflammatory Neuropathy Cause and Treatment (INCAT) disability scale *(Hughes et al*., 2008*)*. These results were supported by statistically significant and clinically relevant improvements in objective clinical measures of maximum grip strength, Medical Research Council (MRC) sum score, and the INCAT sensory sum score *(Hughes et al*., 2008; *Merkies et al*., 2010b*)*.

Privigen is an l-proline-stabilized 10% liquid human IVIG that has demonstrated safety and efficacy as a replacement therapy in primary immunodeficiencies *(Stein et al*., 2009*)* and for immunomodulation in immune thrombocytopenic purpura *(Robak et al*., 2009*)*, and is licensed for those indications in the EU (2008) and the US (2007).

The PRIMA study aimed to confirm the findings of the IVIG-C treatment arm of the ICE study *(Hughes et al*., 2008*)* in an open-label study in both previously treated with IVIG and IVIG treatment-naïve patients.

## Materials and Methods

### Patients, inclusion and exclusion criteria

Males and females aged 18 years or older with definite or probable CIDP, as defined by the EFNS/PNS guidelines *(Joint Task Force of the EFNS and the PNS*, 2010*)*, previously treated or not treated with IVIG, were eligible for this study. Patients were considered not pre-exposed to IVIG (IVIG-naïve) if they had a newly diagnosed CIDP that had developed over at least 2 months, or if their treatment was interrupted for at least 1 year, with a progressive disease that had deteriorated in the last 2 months prior to enrollment. Patients treated during the last 6 months with a stable IVIG dose (maximum dose variation of 20%) at a constant cycle length of 2–6 weeks (maximum cycle length variation of 5 days) were eligible for screening. IVIG-pretreated patients were enrolled in the study if their adjusted INCAT score deteriorated by ≥1 point during the washout period of up to 10 weeks (‘adjusted’ was defined as excluding changes from 0 to 1, or vice versa, solely due to upper limb score).

Patients were excluded if they had been diagnosed with multifocal motor neuropathy with conduction block, monoclonal gammopathy of undetermined significance associated with anti-MAG IgM antibodies, distal acquired demyelinating symmetric neuropathy, or any disease that may have caused similar symptoms or may have interfered with the treatment or with the outcome assessments of the study. Other exclusion criteria were abnormal laboratory parameters (creatinine >1.5 times the upper limit of normal [ULN], lactate dehydrogenase >1.5 times ULN, C-reactive protein >60 mg/dl, hemoglobin <10 g/dl), plasma exchange 3 months prior to enrollment, treatment with immunomodulatory agents other than corticosteroids, methotrexate, or azathioprine within 6 months before enrollment, or with rituximab 12 months before enrollment.

This study was conducted in accordance with the International Conference on Harmonization Good Clinical Practice (ICH GCP) guidelines, and the Declaration of Helsinki (version of 1996). The study protocol and all other study-related documents were reviewed and approved by the local Independent Ethics Committees. Written informed consent was obtained from all patients before the start of the study.

### Study design

This prospective, multicenter, open-label, single-arm Phase III study was designed to investigate the efficacy and safety of Privigen in CIDP.

The regular IVIG treatment of IVIG-pretreated patients was interrupted temporally for up to 10 weeks until disease deterioration occurred (≥1 adjusted INCAT points) that allowed eligibility. All eligible patients received a Privigen induction dose (2 g/kg bw) over 2–5 days, followed by up to seven infusions of 1 g/kg bw at 3-week intervals. Dose reductions were allowed, if medically indicated. Allowed infusion rates ranged from 0.5 mg/kg/min to 8.0 mg/kg/min.

Allowed concomitant therapy included corticosteroids, methotrexate, or azathioprine for CIDP treatment if dosage and frequency of these treatments were stable during the 3 months prior to enrollment and during the study.

### Assessment tools

The outcome measures for this study were selected based on recommendations from previous workshops on outcome assessments in inflammatory neuropathies *(Merkies and Lauria*, 2006; *Lunn et al*., 2008*)*.

The *INCAT disability scale* was chosen as the primary outcome, as this scale had demonstrated good responsiveness in CIDP *(Hughes et al*., 2001, 2008*)*. The INCAT disability scale comprises a practical and functional description of the performance efficiency of the arms and legs in a checklist form suitable for semi-standardized interviewing of patients. Daily arm activities such as dressing the upper part of the body, doing and undoing buttons and zippers, washing or brushing hair, and handling coins are scored as being ‘not affected’, ‘affected but not prevented’, or ‘prevented’. The leg scale measures problems regarding walking, taking into account the use of aids. The *INCAT score* ranges from 0 (no signs of disability) to 10 (most severe disability).

An adapted version of the *MRC sum score* was calculated by summing up the MRC grades (integers ranging 0–5) of the following eight muscle pairs on each side: upper arm abductors, elbow flexors, wrist extensors, first dorsal interosseos, hip flexors, knee extensors, foot dorsal flexors, and extensor hallucis longus. The score ranges from 0 (total paralysis) to 80 (normal strength) *(Kleyweg et al*., 1991*)*.

Grip strength was assessed for both hands using the *Vigorimeter* (Martin, Tuttlingen, Germany), an instrument that has been used to measure grip strength, and has fast and sustained responsiveness in CIDP *(Funfgeld*, 1966; *Hughes et al*., 2008; *Vanhoutte et al*., 2013*)*. The pressure in the medium size bulb is registered by a manometer via a rubber junction tube and is expressed in kilopascals (kPa). The peak (maximum) values of grip strength achieved during each measurement were recorded as maximum grip strength.

All selected centers were uniformly trained by an expert clinimetrician (I. S. J. M.) aiming to standardize the application of selected outcome measures for this study. Each participating center was provided with a research manual containing assessment instructions for the selected outcome measures.

### Efficacy and safety evaluation

The primary efficacy endpoint was the responder rate by the adjusted INCAT score. The INCAT score was measured at baseline and every 3 weeks thereafter, until completion *(Hughes et al*., 2001*)*. The adjustment was introduced when comparing two INCAT scores of the same patient to correct for improvement in the upper limbs only: a change from 1 to 0 or from 0 to 1 solely due to upper limbs score was not considered to be clinically relevant. Responders were defined as patients with a clinically meaningful improvement (decrease of ≥1 point in the adjusted INCAT score) between baseline and completion (Week 25) or the last study visit in case of premature discontinuation.

Secondary efficacy endpoints included time to first clinically meaningful improvement either by adjusted INCAT score (a decrease of ≥1 point) or MRC sum score (an improvement of ≥3 points), and change from baseline in adjusted INCAT score, maximum grip strength of the dominant hand and MRC sum score. All secondary endpoints were assessed at baseline and every 3 weeks thereafter.

Safety endpoints were adverse event (AE) rate per infusion, severity, and relatedness to study medication, vital signs during infusion, and changes in laboratory parameters compared to baseline. Information about AEs and serious AEs (SAEs) was continuously recorded by the investigator in the patient's electronic case report form starting from the time of written informed consent until the completion visit or the last study visit in case of premature discontinuation. All AEs were followed up until resolution or until being recognized as a permanent condition.

Vital signs including systolic and diastolic blood pressure, heart rate, and body temperature were measured repeatedly before, during and after the infusion. Blood samples for measuring IgG levels were taken during screening, before and after the infusion on Week 1 (baseline), Week 7, Week 13 and Week 19, and at the completion visit (Week 25).

In keeping with the ethical requirements of the ICH GCP guidelines, this study used the single-arm study design with a predefined success criterion based on a recent placebo-controlled study of IVIG in patients with CIDP in Europe (ICE study) *(Hughes et al*., 2008*)*. The PRIMA study was considered successful if the lower limit of the 2-sided 95% Wilson-Score confidence interval (CI) of the responder rate by the adjusted INCAT score was greater than 35%, based on the responder rate in the placebo arm of the ICE study (21%; 95% Wilson-Score CI: 12.3%–32.8%) *(Hughes et al*., 2008*)*.

### Statistical methodology

The sample size was calculated based on the predefined success criterion. Due to the lower number of IVIG-naïve patients in this study than in the ICE study, a higher Privigen responder rate was assumed (65% vs. 54% in the ICE study). With a sample size of seven patients, the power to obtain a lower limit of the 2-sided 95% Wilson-Score CI of >35% was greater than 90%. To address a potential misspecification of the assumptions, a total of 30 patients were planned to be enrolled to ensure at least 20 evaluable patients.

Efficacy was determined in the intention-to-treat (ITT) analysis that was based on the full analysis set (FAS), defined as all patients who received at least one infusion. The per-protocol (PP) analysis was based on the valid cases set that included all FAS patients without any major protocol deviation. The primary efficacy endpoint was based on the lower limit of the 2-sided 95% Wilson-Score CI for a single proportion. For secondary efficacy endpoints, the change from baseline to completion was analyzed using non-parametric Hodges–Lehmann point estimates with the corresponding Tukey CI. The time to first adjusted INCAT response and to first MRC response was analyzed using the Kaplan–Meier method.

The safety analysis was based on the safety data set, which was identical to the FAS. Descriptive statistics (mean, standard deviation [SD], median, minimum, maximum, and 25%–75% quantile) were calculated for all safety variables, including laboratory parameters, vital signs, and serum IgG levels.

## Results

### Patients

**Figure 1:**
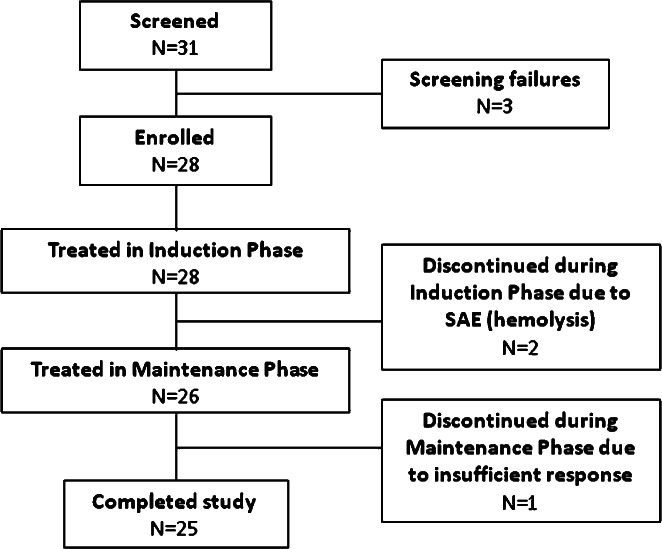
Patient disposition. Of 31 screened patients, 28 were enrolled into the study and received the induction Privigen® dose. A total of three patients discontinued during the induction and maintenance phases, leaving 25 patients who completed the study.

A total of 31 patients were screened at 13 sites in Germany, Belgium, Poland, France, and Finland. The study lasted from December 2010 to November 2011. Three patients did not meet the inclusion criteria, including one patient who did not experience adjusted INCAT score deterioration during the washout period. Twenty-eight patients were enrolled (Table1), of which three patients were discontinued from the study (two patients during the induction period due to SAEs of hemolysis and one patient during the maintenance period due to ‘insufficient response to treatment’), leaving 25 patients who completed the study (Fig. 1).

**Table 1:** Demographic characteristics of patients

Total number of patients	28	
Gender, n (%)		
Male	18	(64.3)
Female	10	(35.7)
Age (years), median (range)	58	(22–79)
Caucasians, n (%)	28	(100)
Body weight (kg), median (range)	83	(50–118)
BMI (kg/m^2^), median (range)	27.9	(18–36)
Height (cm), median (range)	172	(158–195)
Duration of CIDP, n (%)		
≤1 year	9	(32.1)
>1 to ≤2 years	4	(14.3)
>2 to ≤10 years	12	(42.9)
>10 years	3	(10.7)
Baseline disease characteristics		
INCAT score, median (25%–75% quantile)	3.5	(3.0–4.5)
Maximum grip strength (kPa), mean (SD)	66.7	(37.24)
MRC sum score, median (25%–75% quantile)	67.0	(61.5–72.0)
Serum IgG level (mg/dl), mean (SD)	1259.5	(377.47)

Demographic and baseline disease characteristics of all enrolled patients are shown.

BMI, body mass index; CIDP, chronic inflammatory demyelinating polyneuropathy; INCAT, Inflammatory Neuropathy Cause and Treatment disability scale; MRC, Medical Research Council; n, number of patients; SD, standard deviation.

Thirteen patients (46.4%) had been previously treated with IVIG, and 15 patients (53.6%) were IVIG-naïve. The most common (42.9%) time between the CIDP diagnosis and enrollment into the study was 2–10 years (Table1).

All patients received concomitant medication during the study. Most common concomitant medications were analgesics (60.7%), drugs for acid-related disorders (39.3%), agents acting on the renin-angiotensin system (39.3%), and non-steroid anti-inflammatory drugs (NSAIDs) and antirheumatic products (39.3%). Five patients (17.9%) received corticosteroids for systemic use; two patients (7.1%) received azathioprine for immunosuppression.

### Study drug administration

During the induction period, 21 patients (75.0%) received the Privigen induction dose of 2 g/kg bw over 2 days, five patients (17.9%) over 5 days, and two patients (7.1%) over 3 days. The total dose of 2 g/kg bw was split into equal daily amounts.

During the maintenance period, 24 patients (85.7%) received each of the planned seven infusions of 1 g/kg bw on a single day, and one patient received them on 2 consecutive days. The mean of individual patient median infusion rates was 5.89 mg/kg/min, with a range of 1.48 to 8.36 mg/kg/min.

### Efficacy

#### Primary efficacy endpoint

Seventeen (60.7%) of 28 patients in the ITT analysis were responders by the adjusted INCAT score at completion, with the 2-sided 95% Wilson-Score CI of 42.41% to 76.43%, hence meeting the study objectives (Table2). In the PP analysis, 14 (63.6%) of 22 patients were responders, showing an overall responder rate similar to the ITT analysis (95% Wilson-Score CI: 42.95%–80.27%).

**Table 2:** Number of responders by the adjusted INCAT score at completion (ITT)

	All patients	IVIG-pretreated patients	IVIG-naïve patients
Total number of patients	28	13	15
Number of responders, n	17	10	7
Responder rate (%)	60.7	76.9	46.7
Wilson-Score 95% CI of the responder rate (%)	42.4–76.4	49.7–91.8	24.8–69.9

The number and proportion of responders at completion are shown for all patients and patient subgroups in the intention-to-treat (ITT) analysis. Last observation carried forward was used to replace missing values.

CI, confidence interval; INCAT, Inflammatory Neuropathy Cause and Treatment disability scale; IVIG, intravenous immunoglobulin; n, number of patients.

#### Subgroups

IVIG-pretreated patients demonstrated a higher responder rate (76.9%; 10 of 13 patients) than IVIG-naïve patients (46.7%; 7 of 15 patients). Characteristics of the three IVIG-pretreated patients, who did not respond, are shown in Table3.

**Table 3:** Characteristics of IVIG-pretreated non-responders

	Patient I	Patient II	Patient III
Previous IgG dosage (g)	90	160	84
Interval between the last IgG dose and the first Privigen® dose (days)	41	43	44
Privigen induction dose (g)	180	234	168
Privigen maintenance dose (g)	90	117	84
Screening			
INCAT score	4 (2^*^)	2	4
MRC sum score	69	64	60
Maximum grip strength (kPa)	63	160	60
Baseline			
INCAT score	3	3	6
MRC sum score	69	60	54
Maximum grip strength (kPa)	70	150	52
Completion			
INCAT score	3	3	6
MRC sum score	74	75	57
Maximum grip strength (kPa)	110	140	62

Previous intravenous immunoglobulin (IVIG) treatment, Privigen dosage and efficacy measurements are shown for the three IVIG-pretreated patients that did not respond to Privigen after the washout period. Please note that Patient I had no deterioration of the INCAT score during the washout period and therefore should not have been included in the trial. Because he received Privigen treatment during the study, he was included in the intention-to-treat (ITT) analysis, but not in the per-protocol (PP) analysis. INCAT score from a second, unscheduled screening visit of Patient I, which led to inclusion of the patient into the study, is shown in brackets and marked with an asterisk (^*^).

INCAT, Inflammatory Neuropathy Cause and Treatment disability scale; MRC, Medical Research Council.

#### Secondary efficacy endpoints

##### Change from baseline in adjusted INCAT score

**Figure 2:**
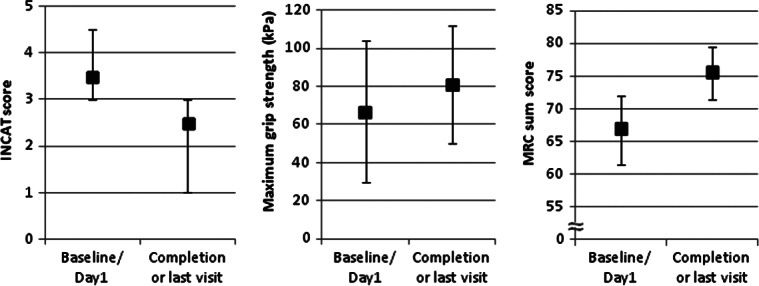
Secondary efficacy outputs have improved from baseline to completion (ITT). The median INCAT score, the mean maximum grip strength and the median MRC sum score are shown for all patients in the ITT analysis. Error bars represent either SD (maximum grip strength) or 25% and 75% quantile (adjusted INCAT score and MRC sum score).

**Figure 3:**
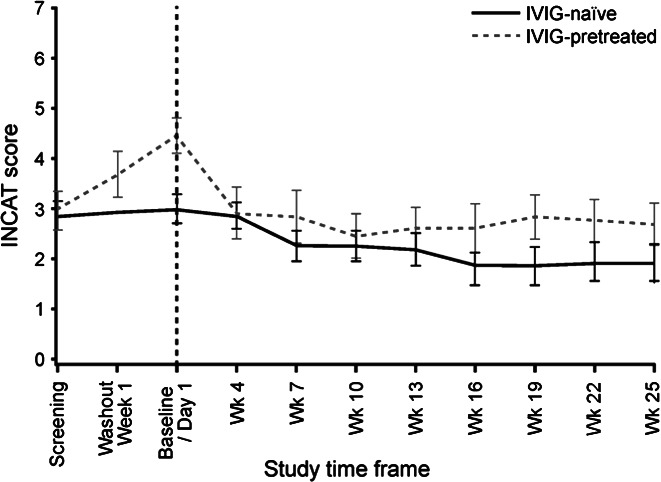
Mean adjusted Inflammatory Neuropathy Cause and Treatment (INCAT) score over time by intravenous immunoglobulin (IVIG)-pretreatment (ITT). The mean adjusted INCAT scores are shown for IVIG-pretreated (dashed gray line) and IVIG-naïve (solid black line) patients in the ITT analysis. Last observation carried forward was used to replace missing values. Error bars represent the standard error of the mean.

The median (25%–75% quantile) adjusted INCAT score improved from 3.5 (3.0–4.5) points at baseline to 2.5 (1.0–3.0) points at completion, a reduction indicating improvement (Fig. 2). The corresponding Hodges–Lehmann estimator indicated a change in the INCAT score of −1.3 points (95% CI: −2.0 to −0.5 points). The mean adjusted INCAT score improved from baseline to completion in both IVIG-pretreated and IVIG-naïve patients (Fig. 3).

##### Time to first adjusted INCAT response

**Figure 4:**
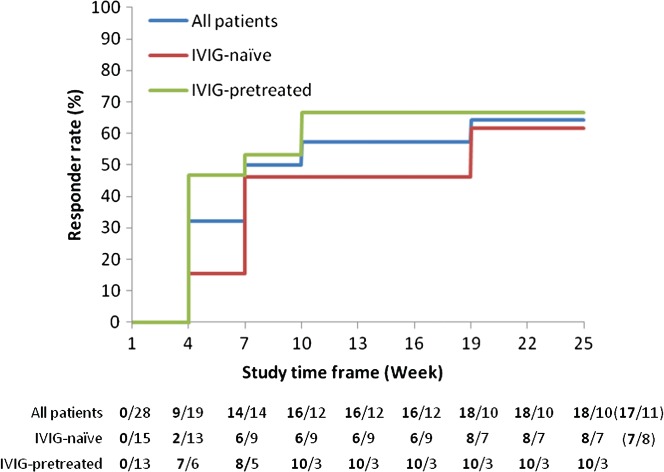
Response to treatment based on adjusted Inflammatory Neuropathy Cause and Treatment (INCAT) score by intravenous immunoglobulin (IVIG)-pretreatment (ITT). Kaplan–Meier analysis of the proportion of responders based on the adjusted INCAT score is shown for all patients (blue), IVIG-naïve (red), and IVIG-pretreated (green) patients in the ITT analysis. The number of patients, who have achieved a response at least once by the time point (n), followed by the number of patients who have not yet responded (N) is indicated as ‘n/N’ below the graph. Please note that this analysis included 1 patient who had a response at Week 19 and Week 22 but not at completion (Week 25), resulting in 18 responders, 17 of which were responders at completion (shown in brackets).

Until completion, 18 of 28 patients responded at least once, with the corresponding Kaplan–Meier estimate of the response probability of 64.3% (95% CI: 46.5%–82.0%). Half of the responders (nine of 18 patients) showed a response after receiving the induction dose, as assessed at Week 4, and seven other patients showed a response by Week 10 (Fig. 4). One patient showed a response at Week 19 and Week 22 but not at completion (Week 25); this patient was therefore considered a non-responder in the primary endpoint analysis. IVIG-pretreated patients demonstrated a shorter median time to first adjusted INCAT response than IVIG-naïve patients (3 weeks vs. 18 weeks; Fig. 4).

##### Maximum grip strength

**Figure 5:**
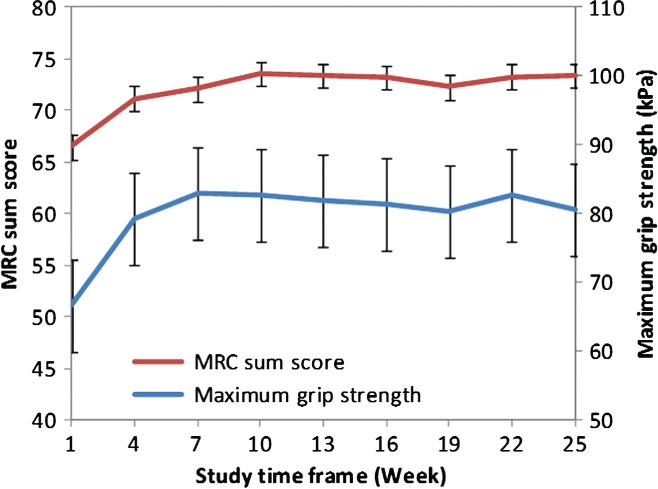
Mean maximum grip strength and mean MRC sum score over time (ITT). The mean maximum grip strength of dominant hand and mean MRC sum score is shown for all patients in the ITT analysis. Error bars represent the standard error of the mean.

The mean (SD) maximum grip strength of the dominant hand increased from 66.7 (37.24) kPa at baseline to 80.9 (31.06) kPa at completion (mean change of 14.0 kPa; 95% CI: 0.79–27.46 kPa; Fig. 2), with a plateau after Week 7 (Fig. 5). Analysis of the maximum grip strength in the non-dominant hand showed similar results (data not shown).

##### Muscle strength (MRC sum score)

The median (25%–75% quantile) MRC sum score increased from 67.0 (61.5–72.0) points at baseline to 75.5 (71.5–79.5) points at completion (Fig. 2), with a plateau after Week 10 (Fig. 5). The corresponding Hodges–Lehmann estimator indicated a change in the MRC score of 6.5 points (95% CI: 4.0–9.5 points).

##### Time to first MRC response

The median (25%–75% quantile) time to first MRC response was 6 weeks (3–9 weeks). On the basis of the MRC sum score, 23 of 28 patients showed a response at completion, with a Kaplan–Meier estimate of the response probability of 84.8%. Thirteen of 15 IVIG-naïve patients and 10 of 13 IVIG-pretreated patients were responders at completion by the MRC score, with the corresponding probabilities of response of 86.7% and 81.5%, respectively.

#### Serum IgG levels

The mean post-infusion serum IgG levels were almost two-fold greater than the pre-infusion IgG levels (Table4). The mean pre- and post-infusion serum IgG levels slightly increased from baseline (1259.5 mg/dl and 2859.2 mg/dl, respectively) to Week 7 (1750.0 mg/dl and 3228.8 mg/dl, respectively) and remained stable until the end of the study. The mean change in IgG levels from pre-infusion to post-infusion was higher, although with greater variability, in patients who had a response than in non-responders (Table4).

**Table 4:** Pre- and post-infusion serum IgG levels (ITT)

	All patients	Responders	Non-responders
Total number of patients	28	17	11
Serum IgG levels (mg/dl)			
Pre-infusion, mean (SD)	1828.4 (547.7)	1881.3 (583.6)	1757.8 (490.9)
Post-infusion, mean (SD)	3363.5 (880.0)	3592.4 (939.7)	3081.8 (711.7)
Change from pre-infusion to post-infusion (mg/dl)			
Mean (SD)	1574.0 (725.4)	1759.0 (758.8)	1342.7 (612.2)
Range	−867 to 3,392	−867 to 3,392	84 to 2,685

The mean and standard deviation (SD) of the pre- and post-infusion serum IgG levels are shown for all patients in the intention-to-treat (ITT) analysis, and for responders and non-responders at completion.

### Safety

#### Overall adverse events

Twenty-two patients (78.6%) experienced a total of 108 AEs, with the overall AE rate of 0.417 AEs per infusion. Ninety-five AEs (88.0%) were mild or moderate in intensity and resolved by the end of the reporting period. The most common AEs were headache, pain in an extremity, hypertension, asthenia, and leukopenia (Table5).

**Table 5:** Most common temporally associated and possibly related AEs (ITT)

	All AEs		Temporally associated AEs (72 hours)		At least possibly related AEs	
	Number of patients (%)	AEs (rate per infusion)	Number of patients (%)	AEs (rate per infusion)	Number of patients (%)	AEs (rate per infusion)
Total number of patients or infusions	28	259	28	259	28	259
Headache	9 (32.1)	20 (0.077)	8 (28.6)	19 (0.073)	8 (28.6)	18 (0.069)
Pain in extremity	6 (21.4)	7 (0.027)	3 (10.7)	3 (0.012)	n/a	n/a
Hypertension	4 (14.3)	6 (0.023)	4 (14.3)	6 (0.023)	4 (14.3)	5 (0.019)
Asthenia	4 (14.3)	4 (0.015)	4 (14.3)	4 (0.015)	4 (14.3)	4 (0.015)
Leukopenia	4 (14.3)	4 (0.015)	0	0	2 (7.1)	2 (0.008)
Nausea	3 (10.7)	3 (0.012)	3 (10.7)	3 (0.012)	3 (10.7)	3 (0.012)
Arthralgia	2 (7.1)	3 (0.012)	1 (3.6)	1 (0.004)	n/a	n/a
Influenza-like illness	2 (7.1)	2 (0.008)	2 (7.1)	2 (0.008)	2 (7.1)	2 (0.008)
Hemolysis	2 (7.1)	2 (0.008)	2 (7.1)	2 (0.008)	2 (7.1)	2 (0.008)
Oropharyngeal pain	2 (7.1)	2 (0.008)	1 (3.6)	1 (0.004)	n/a	n/a
Contusion	2 (7.1)	2 (0.008)	0	0	n/a	n/a
Rash	2 (7.1)	2 (0.008)	0	0	2 (7.1)	2 (0.008)

Numbers of patients and rates per infusion are shown for most common (≥3.5%) adverse events (AEs) for all patients in the intention-to-treat (ITT) analysis. AEs that occurred during or within 72 h after infusion were considered temporally associated. Relatedness of the AEs to study medication was evaluated by the investigators.

n/a, data not available.

#### Temporally associated and related adverse events

Nineteen patients (67.9%) experienced a total of 66 temporally associated AEs that occurred during an infusion or within 72 h after the end of infusion (0.255 temporally associated AEs per infusion). The most common AEs were headache, hypertension, and asthenia (Table5).

Seventeen patients (60.7%) experienced 49 AEs considered at least possibly related to study medication (0.189 related AEs per infusion) including 27 AEs in 13 patients (46.4%) during the induction phase (0.370 related AEs per infusion) and 22 AEs in 11 patients (39.3%) during the maintenance phase (0.118 related AEs per infusion). The most common at least possibly related AEs were headache, hypertension, and asthenia (Table5).

#### Serious adverse events

Four patients (14.3%) experienced a total of four SAEs, of which two cases of hemolysis during the induction phase were assessed as related to study medication (Table6). Both patients were discontinued from the study and recovered without sequelae. No red blood cell transfusion was required for treatment of hemolysis.

**Table 6:** Hemolysis cases

	Patient A	Patient B
Age (years)	56	46
Gender	Female	Male
ABO blood group	AB	A
CIDP diagnosis date	June 2008	June 2009
Previous IVIG	Privigen®	Sandoglobulin®
Last previous dose of IVIG (g)	30	104
Total induction dose (g)	134	208
Induction period (days)	2	2
Induction date	June 2011	March 2011
Concomitant medication	Gabapentin, amlodipine, hydrochlorothiazide, vitamin B complex, enoxaparin	Methylprednisolone, bisoprolol, azathioprine, cotrimoxazole, escitalopram, zolpidem, pantoprazole, trazodone, ibuprofen, paracetamol
Hemolysis symptoms	Headache, jaundice, Hb drop	Fatigue, bilirubinuria, Hb drop
Time between induction and the onset of symptoms (days)	2	2
Hemolysis intensity	Mild	Severe
Time until complete recovery after onset of symptoms (days)	24.5 (11^*^)	33 (21^*^)
Hb level before event (g/dl)	11.8–14.7	15.9
Lowest measured Hb level (g/dl)	9.4	10.5
Time between the onset of symptoms and the lowest measured Hb level (days)	4	4

Characteristics of two patients with hemolysis are shown, including demographics, Privigen dosage, previous and concomitant treatments and hemolysis symptoms and duration. Times between the onset of symptoms and no active signs of hemolysis is shown in brackets and marked with asterisks (^*^).

CIDP, chronic inflammatory demyelinating polyneuropathy; Hb, hemoglobin; IVIG, intravenous immunoglobulin.

Two other SAEs (CIDP deterioration and worsening of chronic sigmoid diverticulitis) were considered not related to Privigen. The SAE of CIDP deterioration occurred 1 day prior to the first Privigen infusion and was, therefore, clearly not related; the patient subsequently responded to study treatment already at Week 4. Both patients completed the study as per protocol and recovered without sequelae.

No deaths occurred during the study.

#### Laboratory tests

Median values and ranges of hematology analytes did not show any relevant changes over time.

Leukopenia was reported in four patients (14.3%) with pre-existing leukopenia at screening, of which two patients also had a medical history of leukopenia. In the first patient, AEs of mild leukopenia were reported at Week 19 (3,520/mm^3^; normal range: 4,800–10,800/mm^3^) and at Week 25 (3,740/mm^3^). These AEs were considered possibly related to Privigen. In the second patient, low leukocyte count at Week 7 (1,730/mm^3^) was reported as an AE of moderate leukopenia and was considered not related to Privigen (considered a measurement artifact in subsequent analysis). The third patient showed low leukocyte count at Week 13 (2,330/mm^3^), which was reported as an AE of mild leukopenia and was considered unlikely related to Privigen. In the fourth patient, AEs of mild leukopenia were reported at Week 13 (2,320/mm^3^) and at Week 19 (3,100/mm^3^). The values were reported as AEs of mild leukopenia possibly related to Privigen.

An AE of moderate hyperbilirubinemia was reported in one patient (3.6%) at Week 25 (2.62 mg/dl; normal range: 0.30–1.20 mg/dl). The AE was considered not related to Privigen.

All laboratory test abnormalities resolved by the final follow-up.

## Discussion

Privigen, administered as a regimen of a 2 g/kg bw induction dose and 1 g/kg bw maintenance doses every 3 weeks, resulted in an adjusted INCAT score-based responder rate of 60.7%. The responder rate was significantly higher than in the historical placebo group (32.8%); hence, the study success criterion was met. This result was supported by consistent improvements in all secondary efficacy outcome measures (adjusted INCAT score, maximum grip strength, and MRC sum score) from baseline to completion. Nine of 17 responders showed a response after receiving the induction dose, as assessed at Week 4. All but one of these patients demonstrated a clinically meaningful improvement by Week 10. Thus, the PRIMA study reported here demonstrated the efficacy of Privigen treatment in patients with CIDP.

The results of the PRIMA study complement the results of the previous studies supporting the efficacy of IVIG in CIDP, including the ICE study *(Hughes et al*., 2001; 2008*)*. Owing to the longer duration than in the ICE study, this study has demonstrated that patients not responding after 6 weeks of IVIG treatment may still show a response at a later time point, in contrast to previously established views *(Latov et al*., 2010*)*. This result suggests that a longer treatment period should be considered before declaring a patient a non-responder to IVIG therapy.

Three of 13 IVIG-pretreated patients did not respond to Privigen after the washout period and re-introduction of the IVIG treatment. Two of these patients had a relevant improvement in grip strength and MRC sum score, but did not return to their screening INCAT score. A probable explanation is that the follow-up time was too short to allow for full INCAT score-based recovery. In addition, one of these patients did not deteriorate during the washout period, indicating a possibility of CIDP becoming inactive after the initial treatment, which is a known reason of no response after re-introduction of the IgG therapy *(McCombe et al*., 1987*)*. The third non-responder patient received a lower IVIG dose than previously given (113 g every 3 weeks vs. 160 g every 3 weeks before the washout period). This result indicates a potential case of insufficient dosing. The proportion of IVIG-naïve responders in this study was slightly lower than in the ICE study (46.7% vs. 51.3%); yet generally in the same range *(Hughes et al*., 2008*)*.

The PRIMA study showed that Privigen treatment was well tolerated by patients with CIDP. Two patients had SAEs of hemolysis that were considered at least possibly related to Privigen, one of which had been on regular Privigen before entering the study. Both SAEs occurred within 2 days after the induction dose of Privigen and resolved after discontinuation of the treatment without further interventions (e.g., no blood transfusion was necessary). Hemolysis is a known, rare side effect of IVIG, with an increased risk in patients receiving high doses of IVIG (such as those used in immunomodulation for CIDP), with non-O blood group and/or an underlying inflammatory state *(Daw et al*., 2008; *Padmore*, 2012*)*. All these risk factors (blood groups A and AB, and a substantially increased IVIG dose compared to previous treatment) were present in both patients with hemolysis. Concomitant cotrimoxazole treatment was considered a possible additional risk factor in one of the patients. A gallstone and concomitant medication (gabapentin and amlodipine) were reported as an alternative causality in the other patient. In a study in 57 patients with immune thrombocytopenia receiving Privigen, two cases of laboratory signs of non-serious hemolytic reactions (anemia and positive direct Coombs' test) were reported that were transient and did not require medical intervention *(Robak et al*., 2009*)*. In an ongoing post-marketing study of Privigen in all registered indications, no cases of hemolysis have been reported in 814 patients *(Otremba et al*., 2012*)*. These findings suggest that patients with risk factors for hemolysis receiving IVIG must be monitored carefully, as this AE can easily be overlooked.

Four episodes of mild leukopenia in two patients with previous medical history of leukopenia were considered at least possibly related to Privigen. IVIG-related cytopenias are known to be often transient and are usually not considered to be clinically significant *(Baxley and Akhtari*, 2011*)*.

One of the limitations of this study was the usage of a historical control group. According to the ICH GCP guidelines, using a placebo control in a second study in the same patient population is not recommended. Considering the availability of placebo data in European patients with CIDP from the ICE study *(Hughes et al*., 2008*)* and the relatively low prevalence of CIDP *(Joint Task Force of the EFNS and the PNS*, 2010*)*, the single-arm study design with a predefined success criterion based on a historical control group was chosen to show the efficacy of Privigen over placebo. The active control design was not used, because showing non-inferiority of Privigen to other products was out of the scope of this study. Any other study design (e.g., head-to-head comparison) would have made it extremely difficult, if not impossible, to recruit the required number of patients. Despite these methodological limitations, the PRIMA study provides sufficient evidence of efficacy and tolerability of Privigen treatment in patients with CIDP.

## Appendix

List of investigators: Belgium: J. L. De Bleecker, AZ St-Lucas, Gent (5 patients); W. Robberecht, UZ Leuven, Leuven (3 patients). Finland: M. Saarela, HUS Meilahti Hospital, Helsinki (3 patients). France: J. Franques, Hôpital de la Timone, Neurologie et Maladies Neuro-Musculaire, Marseille (2 patients); J.-M. Léger, Groupe Hospitalier Pitié-Salpêtrière Unité de Pathologie Neuro-Musculaire, Paris (1 patient); R. Juntas Morales, CHRU Hôpital Gui de Chauliac, Montpellier (1 patient). Germany: C. Sommer, Universitätsklinikum Würzburg, Würzburg (4 patients); A. Nguento, ASKLEPIOS Klinikum Uckermark GmbH, Schwedt (2 patients); J. Schmidt, Universtitätsmedizin Göttingen, Georg-August-Universität, Göttingen (1 patient); Ch. Schrey, Facharzt für Neurologie, Berlin (1 patient). Poland: J. Kamienowski, Dolnośląski Szpital Specjalistyczny, Wrocław (3 patients); Z. Stelmasiak, Samodzielny Publiczny Szpital Kliniczny, Lublin (3 patients); G. Zwolińska, Centrum Neurologii Klinicznej, Kraków (2 patients).
